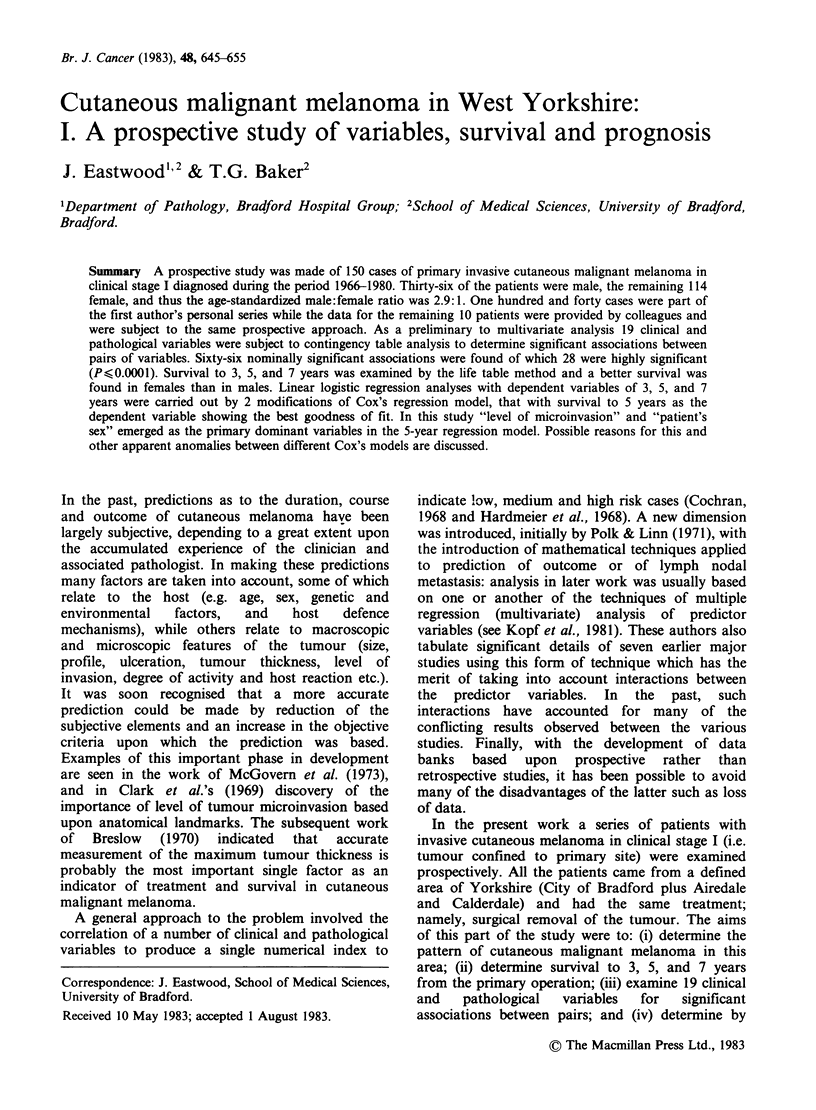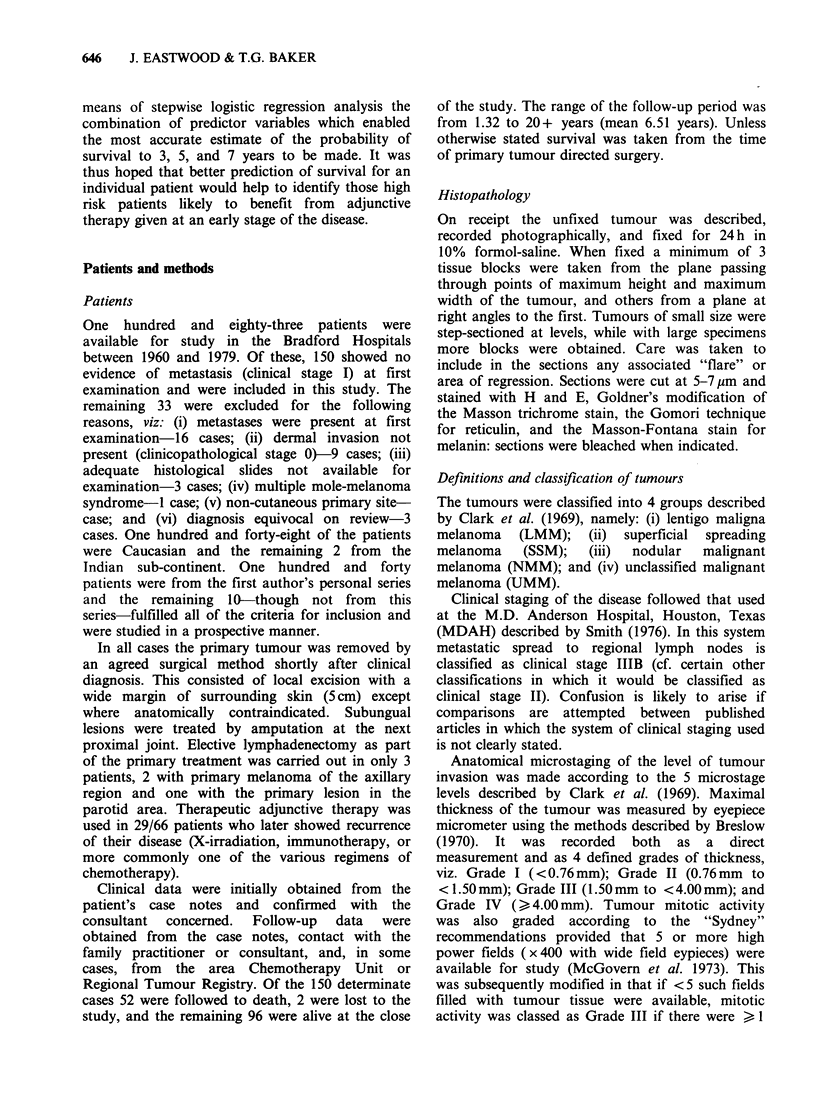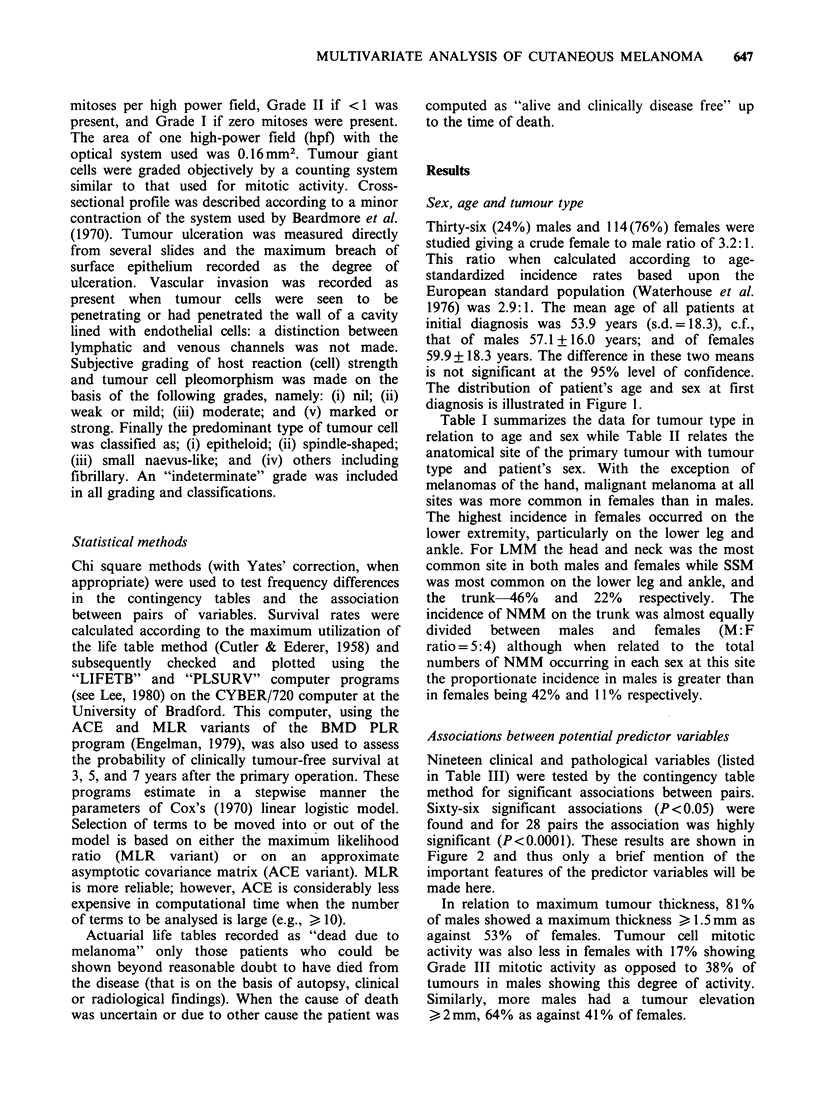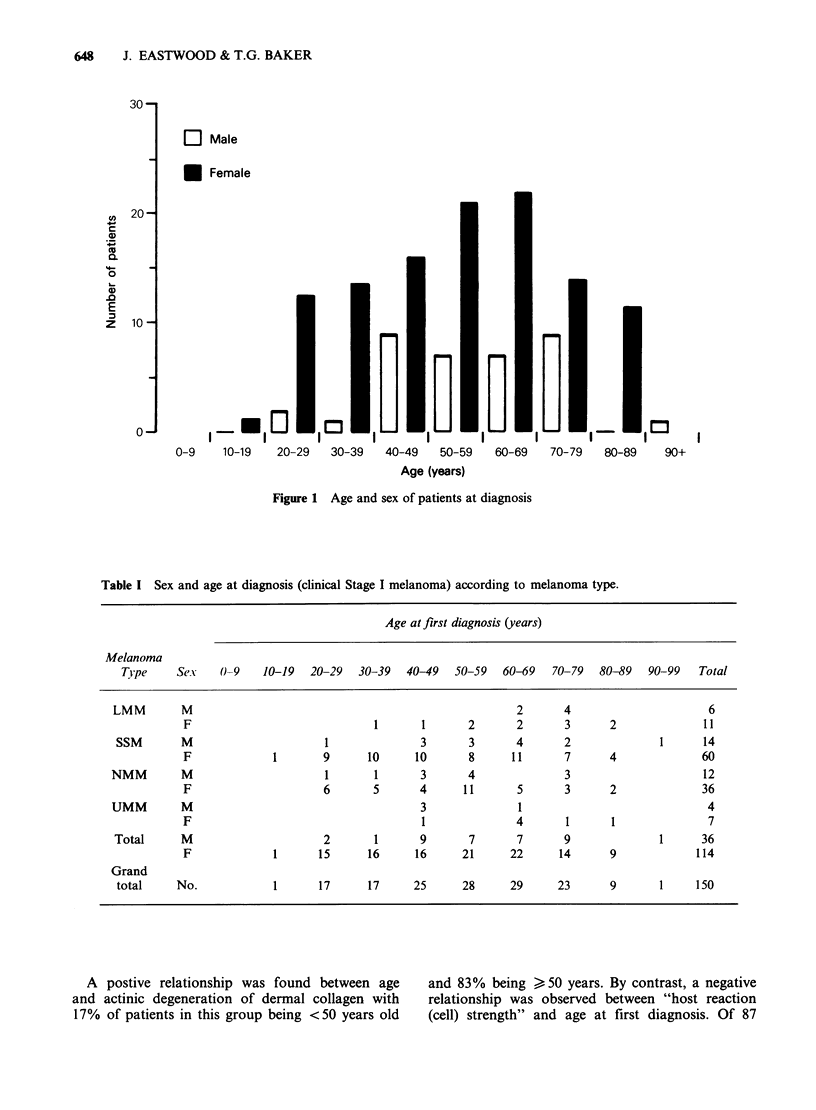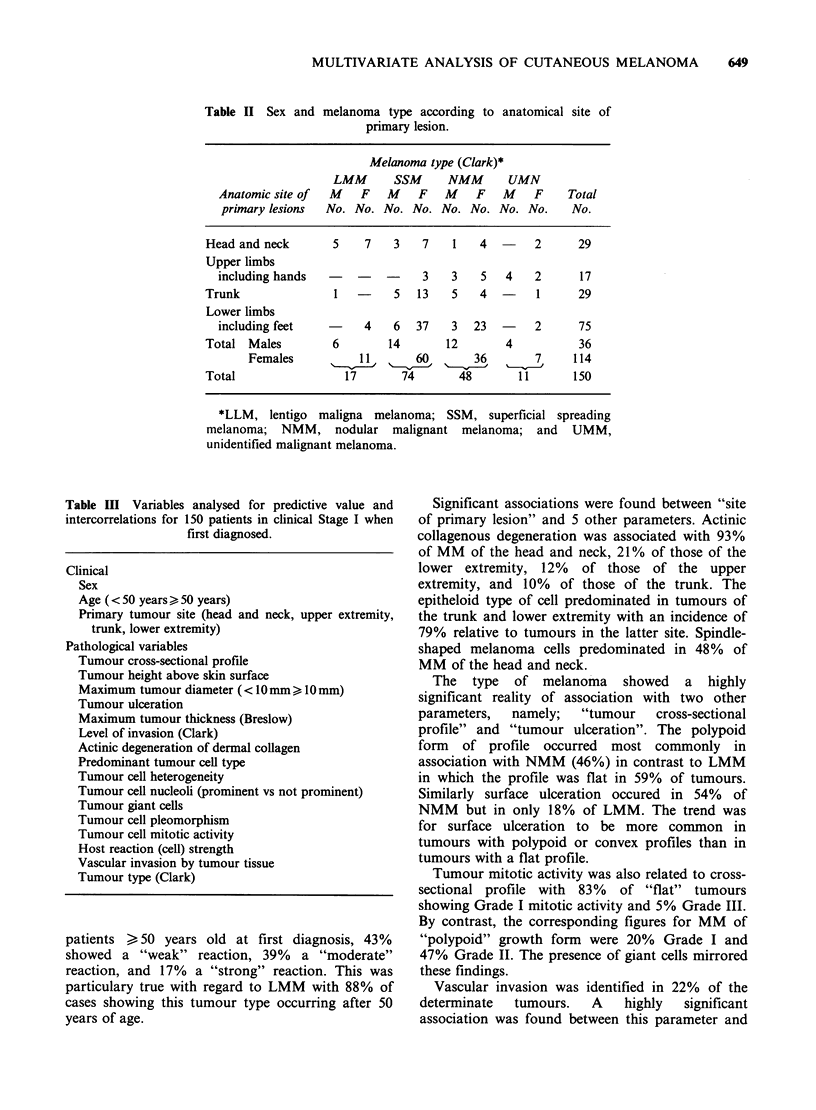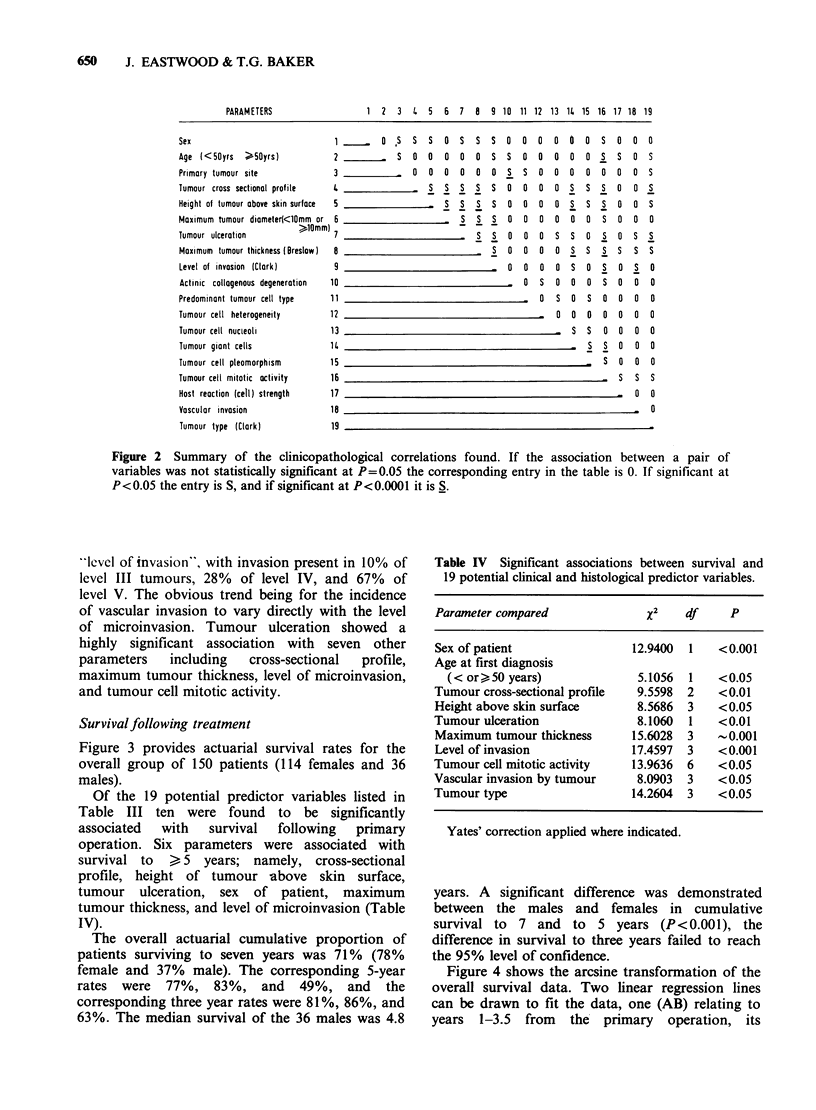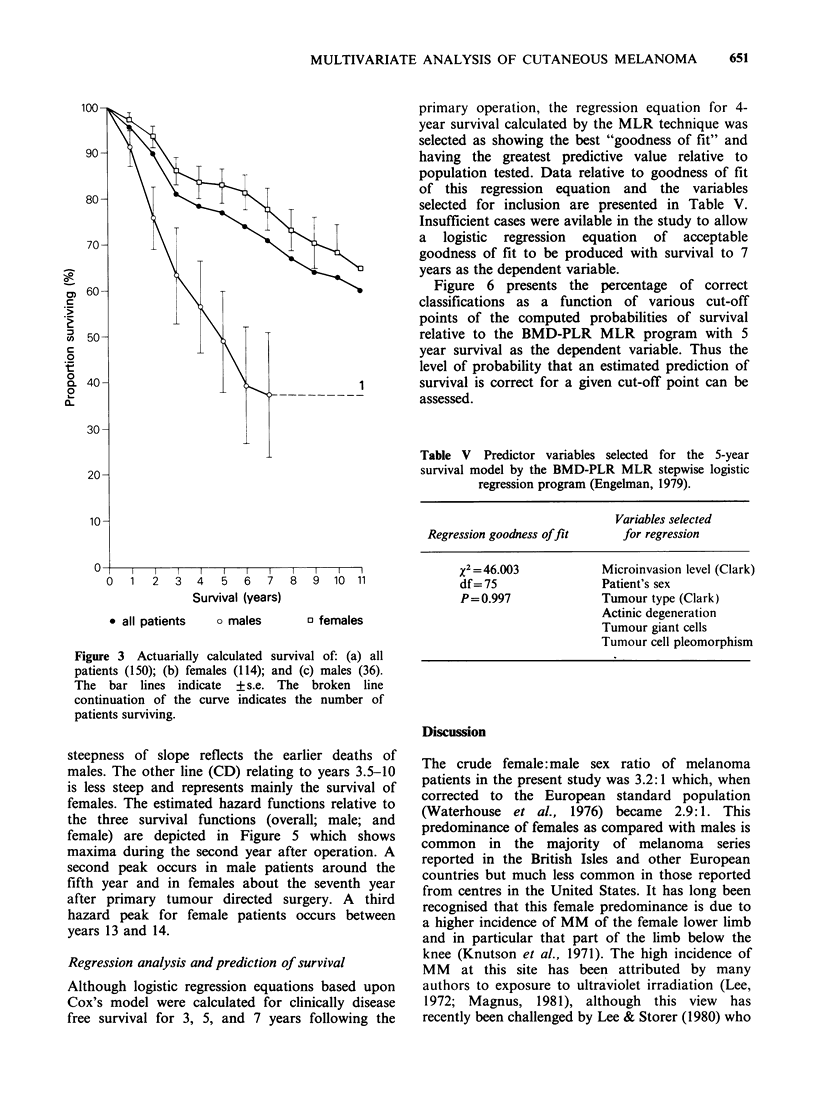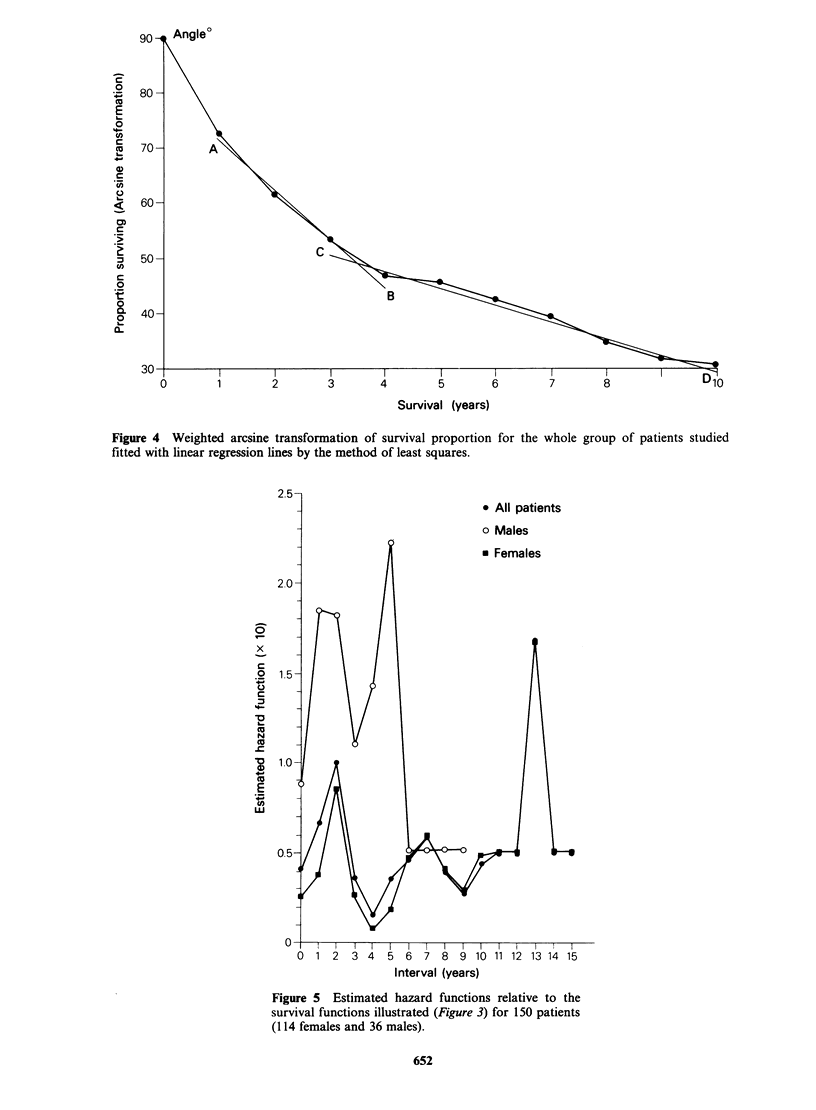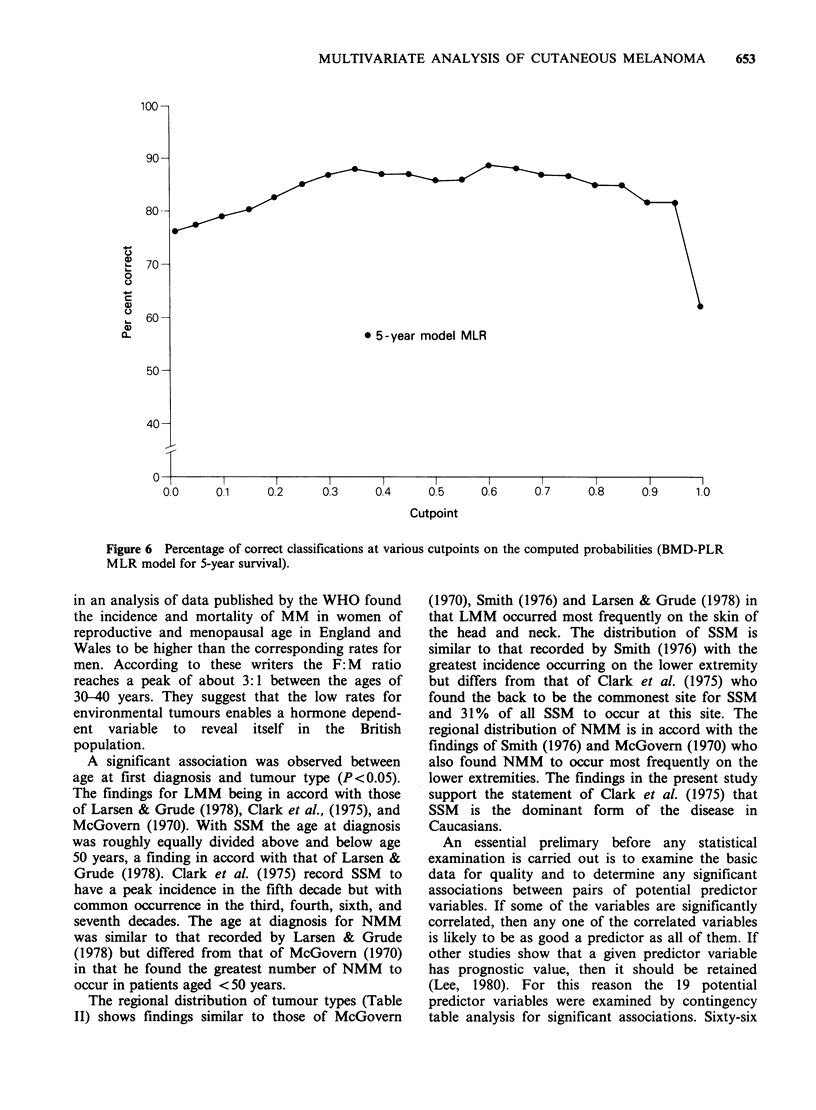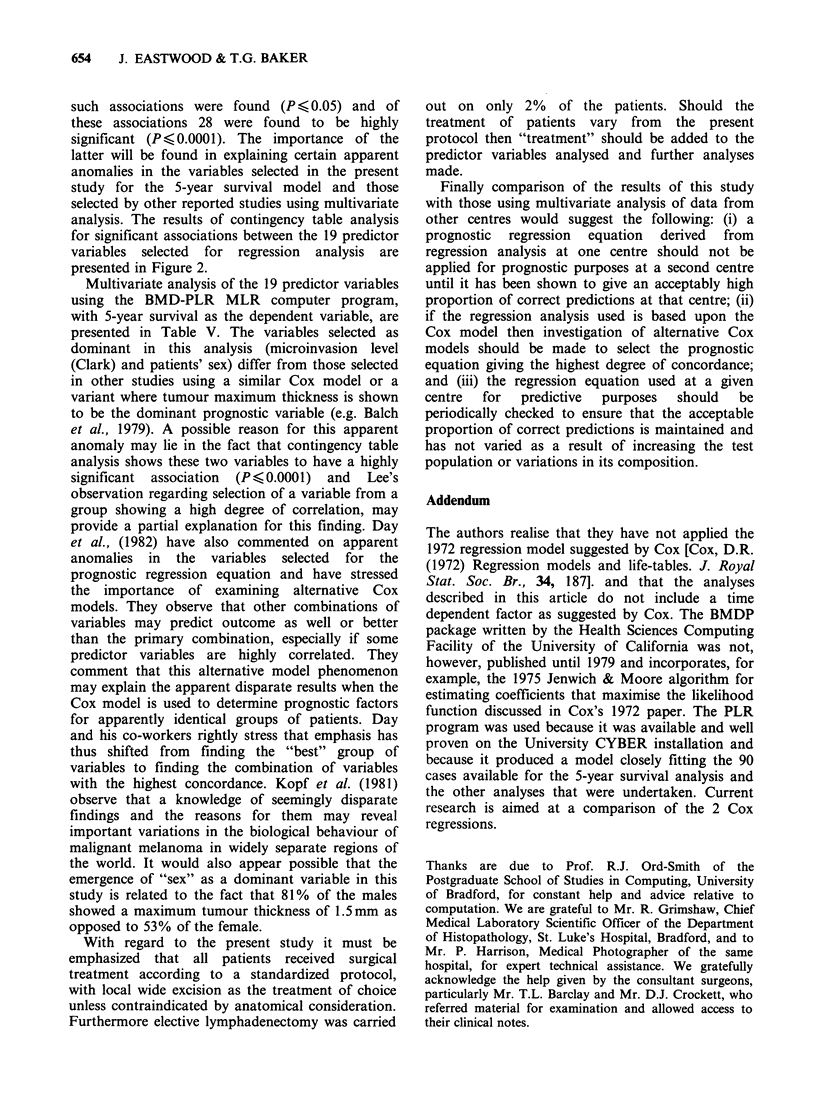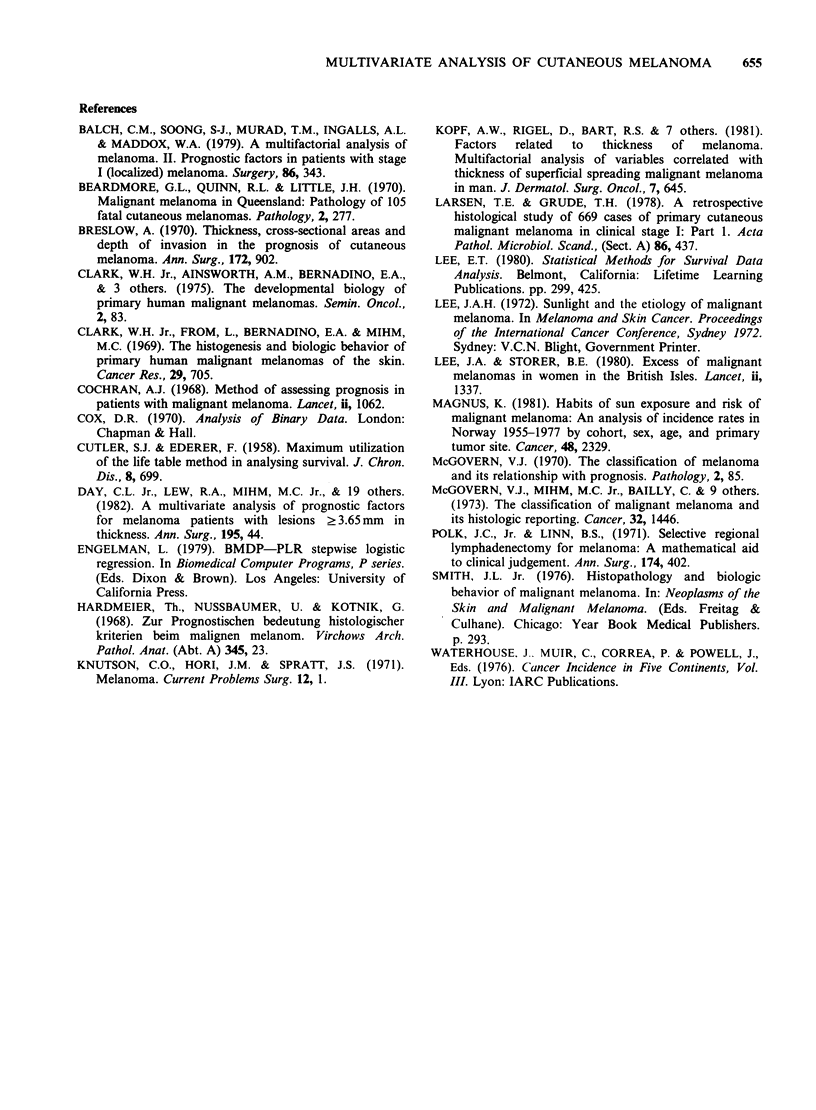# Cutaneous malignant melanoma in West Yorkshire: I. A prospective study of variables, survival and prognosis.

**DOI:** 10.1038/bjc.1983.246

**Published:** 1983-11

**Authors:** J. Eastwood, T. G. Baker

## Abstract

A prospective study was made of 150 cases of primary invasive cutaneous malignant melanoma in clinical stage I diagnosed during the period 1966-1980. Thirty-six of the patients were male, the remaining 114 female, and thus the age-standardized male:female ratio was 2.9:1. One hundred and forty cases were part of the first author's personal series while the data for the remaining 10 patients were provided by colleagues and were subject to the same prospective approach. As a preliminary to multivariate analysis 19 clinical and pathological variables were subject to contingency table analysis to determine significant associations between pairs of variables. Sixty-six nominally significant associations were found of which 28 were highly significant (P less than or equal to 0.0001). Survival to 3, 5, and 7 years was examined by the life table method and a better survival was found in females than in males. Linear logistic regression analyses with dependent variables of 3, 5, and 7 years were carried out by 2 modifications of Cox's regression model, that with survival to 5 years as the dependent variable showing the best goodness of fit. In this study "level of microinvasion" and "patient's sex" emerged as the primary dominant variables in the 5-year regression model. Possible reasons for this and other apparent anomalies between different Cox's models are discussed.


*Br. J. Cancer* (1983), **48**, 645–655

### Cutaneous malignant melanoma in West Yorkshire: I. A prospective study of variables, survival and prognosis

J. Eastwood[1,2] & T.G. Baker[2]

[1]*Department of Pathology, Bradford Hospital Group;* [2]*School of Medical Sciences, University of Bradford, Bradford.*

**Summary** A prospective study was made of 150 cases of primary invasive cutaneous malignant melanoma in clinical stage I diagnosed during the period 1966–1980. Thirty-six of the patients were male, the remaining 114 female, and thus the age-standardized male:female ratio was 2.9:1. One hundred and forty cases were part of the first author's personal series while the data for the remaining 10 patients were provided by colleagues and were subject to the same prospective approach. As a preliminary to multivariate analysis 19 clinical and pathological variables were subject to contingency table analysis to determine significant associations between pairs of variables. Sixty-six nominally significant associations were found of which 28 were highly significant ($P \leqslant 0.0001$). Survival to 3, 5, and 7 years was examined by the life table method and a better survival was found in females than in males. Linear logistic regression analyses with dependent variables of 3, 5, and 7 years were carried out by 2 modifications of Cox's regression model, that with survival to 5 years as the dependent variable showing the best goodness of fit. In this study "level of microinvasion" and "patient's sex" emerged as the primary dominant variables in the 5-year regression model. Possible reasons for this and other apparent anomalies between different Cox's models are discussed.

In the past, predictions as to the duration, course and outcome of cutaneous melanoma have been largely subjective, depending to a great extent upon the accumulated experience of the clinician and associated pathologist. In making these predictions many factors are taken into account, some of which relate to the host (e.g. age, sex, genetic and environmental factors, and host defence mechanisms), while others relate to macroscopic and microscopic features of the tumour (size, profile, ulceration, tumour thickness, level of invasion, degree of activity and host reaction etc.). It was soon recognised that a more accurate prediction could be made by reduction of the subjective elements and an increase in the objective criteria upon which the prediction was based. Examples of this important phase in development are seen in the work of McGovern *et al.* (1973), and in Clark *et al.*'s (1969) discovery of the importance of level of tumour microinvasion based upon anatomical landmarks. The subsequent work of Breslow (1970) indicated that accurate measurement of the maximum tumour thickness is probably the most important single factor as an indicator of treatment and survival in cutaneous malignant melanoma.

A general approach to the problem involved the correlation of a number of clinical and pathological variables to produce a single numerical index to indicate low, medium and high risk cases (Cochran, 1968 and Hardmeier *et al.*, 1968). A new dimension was introduced, initially by Polk & Linn (1971), with the introduction of mathematical techniques applied to prediction of outcome or of lymph nodal metastasis: analysis in later work was usually based on one or another of the techniques of multiple regression (multivariate) analysis of predictor variables (see Kopf *et al.*, 1981). These authors also tabulate significant details of seven earlier major studies using this form of technique which has the merit of taking into account interactions between the predictor variables. In the past, such interactions have accounted for many of the conflicting results observed between the various studies. Finally, with the development of data banks based upon prospective rather than retrospective studies, it has been possible to avoid many of the disadvantages of the latter such as loss of data.

In the present work a series of patients with invasive cutaneous melanoma in clinical stage I (i.e. tumour confined to primary site) were examined prospectively. All the patients came from a defined area of Yorkshire (City of Bradford plus Airedale and Calderdale) and had the same treatment; namely, surgical removal of the tumour. The aims of this part of the study were to: (i) determine the pattern of cutaneous malignant melanoma in this area; (ii) determine survival to 3, 5, and 7 years from the primary operation; (iii) examine 19 clinical and pathological variables for significant associations between pairs; and (iv) determine by

Correspondence: J. Eastwood, School of Medical Sciences, University of Bradford.

Received 10 May 1983; accepted 1 August 1983.

© The Macmillan Press Ltd., 1983

means of stepwise logistic regression analysis the combination of predictor variables which enabled the most accurate estimate of the probability of survival to 3, 5, and 7 years to be made. It was thus hoped that better prediction of survival for an individual patient would help to identify those high risk patients likely to benefit from adjunctive therapy given at an early stage of the disease.

### Patients and methods

### *Patients*

One hundred and eighty-three patients were available for study in the Bradford Hospitals between 1960 and 1979. Of these, 150 showed no evidence of metastasis (clinical stage I) at first examination and were included in this study. The remaining 33 were excluded for the following reasons, *viz:* (i) metastases were present at first examination—16 cases; (ii) dermal invasion not present (clinicopathological stage 0)—9 cases; (iii) adequate histological slides not available for examination—3 cases; (iv) multiple mole-melanoma syndrome—1 case; (v) non-cutaneous primary site—case; and (vi) diagnosis equivocal on review—3 cases. One hundred and forty-eight of the patients were Caucasian and the remaining 2 from the Indian sub-continent. One hundred and forty patients were from the first author's personal series and the remaining 10—though not from this series—fulfilled all of the criteria for inclusion and were studied in a prospective manner.

In all cases the primary tumour was removed by an agreed surgical method shortly after clinical diagnosis. This consisted of local excision with a wide margin of surrounding skin (5 cm) except where anatomically contraindicated. Subungual lesions were treated by amputation at the next proximal joint. Elective lymphadenectomy as part of the primary treatment was carried out in only 3 patients, 2 with primary melanoma of the axillary region and one with the primary lesion in the parotid area. Therapeutic adjunctive therapy was used in 29/66 patients who later showed recurrence of their disease (X-irradiation, immunotherapy, or more commonly one of the various regimens of chemotherapy).

Clinical data were initially obtained from the patient's case notes and confirmed with the consultant concerned. Follow-up data were obtained from the case notes, contact with the family practitioner or consultant, and, in some cases, from the area Chemotherapy Unit or Regional Tumour Registry. Of the 150 determinate cases 52 were followed to death, 2 were lost to the study, and the remaining 96 were alive at the close of the study. The range of the follow-up period was from 1.32 to 20+ years (mean 6.51 years). Unless otherwise stated survival was taken from the time of primary tumour directed surgery.

### *Histopathology*

On receipt the unfixed tumour was described, recorded photographically, and fixed for 24 h in 10% formol-saline. When fixed a minimum of 3 tissue blocks were taken from the plane passing through points of maximum height and maximum width of the tumour, and others from a plane at right angles to the first. Tumours of small size were step-sectioned at levels, while with large specimens more blocks were obtained. Care was taken to include in the sections any associated "flare" or area of regression. Sections were cut at 5–7 $\mu$m and stained with H and E, Goldner's modification of the Masson trichrome stain, the Gomori technique for reticulin, and the Masson-Fontana stain for melanin: sections were bleached when indicated.

### *Definitions and classification of tumours*

The tumours were classified into 4 groups described by Clark *et al.* (1969), namely: (i) lentigo maligna melanoma (LMM); (ii) superficial spreading melanoma (SSM); (iii) nodular malignant melanoma (NMM); and (iv) unclassified malignant melanoma (UMM).

Clinical staging of the disease followed that used at the M.D. Anderson Hospital, Houston, Texas (MDAH) described by Smith (1976). In this system metastatic spread to regional lymph nodes is classified as clinical stage IIIB (cf. certain other classifications in which it would be classified as clinical stage II). Confusion is likely to arise if comparisons are attempted between published articles in which the system of clinical staging used is not clearly stated.

Anatomical microstaging of the level of tumour invasion was made according to the 5 microstage levels described by Clark *et al.* (1969). Maximal thickness of the tumour was measured by eyepiece micrometer using the methods described by Breslow (1970). It was recorded both as a direct measurement and as 4 defined grades of thickness, viz. Grade I ($<$0.76 mm); Grade II (0.76 mm to $<$1.50 mm); Grade III (1.50 mm to $<$4.00 mm); and Grade IV ($\geqslant$4.00 mm). Tumour mitotic activity was also graded according to the "Sydney" recommendations provided that 5 or more high power fields ($\times$400 with wide field eypieces) were available for study (McGovern *et al.* 1973). This was subsequently modified in that if $<$5 such fields filled with tumour tissue were available, mitotic activity was classed as Grade III if there were $\geqslant$1

mitoses per high power field, Grade II if <1 was present, and Grade I if zero mitoses were present. The area of one high-power field (hpf) with the optical system used was 0.16 $mm^2$. Tumour giant cells were graded objectively by a counting system similar to that used for mitotic activity. Cross-sectional profile was described according to a minor contraction of the system used by Beardmore *et al.* (1970). Tumour ulceration was measured directly from several slides and the maximum breach of surface epithelium recorded as the degree of ulceration. Vascular invasion was recorded as present when tumour cells were seen to be penetrating or had penetrated the wall of a cavity lined with endothelial cells: a distinction between lymphatic and venous channels was not made. Subjective grading of host reaction (cell) strength and tumour cell pleomorphism was made on the basis of the following grades, namely: (i) nil; (ii) weak or mild; (iii) moderate; and (v) marked or strong. Finally the predominant type of tumour cell was classified as; (i) epitheloid; (ii) spindle-shaped; (iii) small naevus-like; and (iv) others including fibrillary. An "indeterminate" grade was included in all grading and classifications.

### *Statistical methods*

Chi square methods (with Yates' correction, when appropriate) were used to test frequency differences in the contingency tables and the association between pairs of variables. Survival rates were calculated according to the maximum utilization of the life table method (Cutler & Ederer, 1958) and subsequently checked and plotted using the "LIFETB" and "PLSURV" computer programs (see Lee, 1980) on the CYBER/720 computer at the University of Bradford. This computer, using the ACE and MLR variants of the BMD PLR program (Engelman, 1979), was also used to assess the probability of clinically tumour-free survival at 3, 5, and 7 years after the primary operation. These programs estimate in a stepwise manner the parameters of Cox's (1970) linear logistic model. Selection of terms to be moved into or out of the model is based on either the maximum likelihood ratio (MLR variant) or on an approximate asymptotic covariance matrix (ACE variant). MLR is more reliable; however, ACE is considerably less expensive in computational time when the number of terms to be analysed is large (e.g., $\geqslant 10$).

Actuarial life tables recorded as "dead due to melanoma" only those patients who could be shown beyond reasonable doubt to have died from the disease (that is on the basis of autopsy, clinical or radiological findings). When the cause of death was uncertain or due to other cause the patient was computed as "alive and clinically disease free" up to the time of death.

### Results

### *Sex, age and tumour type*

Thirty-six (24%) males and 114 (76%) females were studied giving a crude female to male ratio of 3.2:1. This ratio when calculated according to age-standardized incidence rates based upon the European standard population (Waterhouse *et al.* 1976) was 2.9:1. The mean age of all patients at initial diagnosis was 53.9 years (s.d. = 18.3), c.f., that of males $57.1 \pm 16.0$ years; and of females $59.9 \pm 18.3$ years. The difference in these two means is not significant at the 95% level of confidence. The distribution of patient's age and sex at first diagnosis is illustrated in Figure 1.

Table I summarizes the data for tumour type in relation to age and sex while Table II relates the anatomical site of the primary tumour with tumour type and patient's sex. With the exception of melanomas of the hand, malignant melanoma at all sites was more common in females than in males. The highest incidence in females occurred on the lower extremity, particularly on the lower leg and ankle. For LMM the head and neck was the most common site in both males and females while SSM was most common on the lower leg and ankle, and the trunk—46% and 22% respectively. The incidence of NMM on the trunk was almost equally divided between males and females (M:F ratio = 5:4) although when related to the total numbers of NMM occurring in each sex at this site the proportionate incidence in males is greater than in females being 42% and 11% respectively.

### *Associations between potential predictor variables*

Nineteen clinical and pathological variables (listed in Table III) were tested by the contingency table method for significant associations between pairs. Sixty-six significant associations ($P<0.05$) were found and for 28 pairs the association was highly significant ($P<0.0001$). These results are shown in Figure 2 and thus only a brief mention of the important features of the predictor variables will be made here.

In relation to maximum tumour thickness, 81% of males showed a maximum thickness $\geqslant 1.5$ mm as against 53% of females. Tumour cell mitotic activity was also less in females with 17% showing Grade III mitotic activity as opposed to 38% of tumours in males showing this degree of activity. Similarly, more males had a tumour elevation $\geqslant 2$ mm, 64% as against 41% of females.

**Figure 1** Age and sex of patients at diagnosis

**Table I** Sex and age at diagnosis (clinical Stage I melanoma) according to melanoma type.

| Melanoma Type | Sex | Age at first diagnosis (years) 0-9 | 10-19 | 20-29 | 30-39 | 40-49 | 50-59 | 60-69 | 70-79 | 80-89 | 90-99 | Total |
|---|---|---|---|---|---|---|---|---|---|---|---|---|
| LMM | M | | | | | | | 2 | 4 | | | 6 |
| | F | | | | 1 | 1 | 2 | 2 | 3 | 2 | | 11 |
| SSM | M | | | 1 | | 3 | 3 | 4 | 2 | | 1 | 14 |
| | F | | 1 | 9 | 10 | 10 | 8 | 11 | 7 | 4 | | 60 |
| NMM | M | | | 1 | 1 | 3 | 4 | | 3 | | | 12 |
| | F | | | 6 | 5 | 4 | 11 | 5 | 3 | 2 | | 36 |
| UMM | M | | | | | 3 | | 1 | | | | 4 |
| | F | | | | | 1 | | 4 | 1 | 1 | | 7 |
| Total | M | | | 2 | 1 | 9 | 7 | 7 | 9 | | 1 | 36 |
| | F | | 1 | 15 | 16 | 16 | 21 | 22 | 14 | 9 | | 114 |
| Grand total | No. | | 1 | 17 | 17 | 25 | 28 | 29 | 23 | 9 | 1 | 150 |

A postive relationship was found between age and actinic degeneration of dermal collagen with 17% of patients in this group being <50 years old and 83% being ≥50 years. By contrast, a negative relationship was observed between "host reaction (cell) strength" and age at first diagnosis. Of 87

**Table II** Sex and melanoma type according to anatomical site of primary lesion.

| | *Melanoma type (Clark)** | | | | | | | | |
|---|---|---|---|---|---|---|---|---|---|
| | *LMM* | | *SSM* | | *NMM* | | *UMN* | | |
| *Anatomic site of primary lesions* | *M No.* | *F No.* | *M No.* | *F No.* | *M No.* | *F No.* | *M No.* | *F No.* | *Total No.* |
| Head and neck | 5 | 7 | 3 | 7 | 1 | 4 | — | 2 | 29 |
| Upper limbs including hands | — | — | — | 3 | 3 | 5 | 4 | 2 | 17 |
| Trunk | 1 | — | 5 | 13 | 5 | 4 | — | 1 | 29 |
| Lower limbs including feet | — | 4 | 6 | 37 | 3 | 23 | — | 2 | 75 |
| Total Males | 6 | | 14 | | 12 | | 4 | | 36 |
| Females | | 11 | | 60 | | 36 | | 7 | 114 |
| Total | 17 | | 74 | | 48 | | 11 | | 150 |

*LLM, lentigo maligna melanoma; SSM, superficial spreading melanoma; NMM, nodular malignant melanoma; and UMM, unidentified malignant melanoma.

**Table III** Variables analysed for predictive value and intercorrelations for 150 patients in clinical Stage I when first diagnosed.

Clinical
- Sex
- Age (<50 years≥50 years)
- Primary tumour site (head and neck, upper extremity, trunk, lower extremity)

Pathological variables
- Tumour cross-sectional profile
- Tumour height above skin surface
- Maximum tumour diameter (<10 mm≥10 mm)
- Tumour ulceration
- Maximum tumour thickness (Breslow)
- Level of invasion (Clark)
- Actinic degeneration of dermal collagen
- Predominant tumour cell type
- Tumour cell heterogeneity
- Tumour cell nucleoli (prominent vs not prominent)
- Tumour giant cells
- Tumour cell pleomorphism
- Tumour cell mitotic activity
- Host reaction (cell) strength
- Vascular invasion by tumour tissue
- Tumour type (Clark)

patients ≥50 years old at first diagnosis, 43% showed a "weak" reaction, 39% a "moderate" reaction, and 17% a "strong" reaction. This was particulary true with regard to LMM with 88% of cases showing this tumour type occurring after 50 years of age.

Significant associations were found between "site of primary lesion" and 5 other parameters. Actinic collagenous degeneration was associated with 93% of MM of the head and neck, 21% of those of the lower extremity, 12% of those of the upper extremity, and 10% of those of the trunk. The epitheloid type of cell predominated in tumours of the trunk and lower extremity with an incidence of 79% relative to tumours in the latter site. Spindle-shaped melanoma cells predominated in 48% of MM of the head and neck.

The type of melanoma showed a highly significant reality of association with two other parameters, namely; "tumour cross-sectional profile" and "tumour ulceration". The polypoid form of profile occurred most commonly in association with NMM (46%) in contrast to LMM in which the profile was flat in 59% of tumours. Similarly surface ulceration occured in 54% of NMM but in only 18% of LMM. The trend was for surface ulceration to be more common in tumours with polypoid or convex profiles than in tumours with a flat profile.

Tumour mitotic activity was also related to cross-sectional profile with 83% of "flat" tumours showing Grade I mitotic activity and 5% Grade III. By contrast, the corresponding figures for MM of "polypoid" growth form were 20% Grade I and 47% Grade II. The presence of giant cells mirrored these findings.

Vascular invasion was identified in 22% of the determinate tumours. A highly significant association was found between this parameter and

| PARAMETERS | | 1 | 2 | 3 | 4 | 5 | 6 | 7 | 8 | 9 | 10 | 11 | 12 | 13 | 14 | 15 | 16 | 17 | 18 | 19 |
|---|---|---|---|---|---|---|---|---|---|---|---|---|---|---|---|---|---|---|---|---|
| Sex | 1 | — | 0 | S | S | S | 0 | S | S | S | 0 | 0 | 0 | 0 | 0 | 0 | S | 0 | 0 | 0 |
| Age (<50yrs ⩾50yrs) | 2 | — | — | S | 0 | 0 | 0 | 0 | 0 | S | S | 0 | 0 | 0 | 0 | 0 | S̲ | S | 0 | S |
| Primary tumour site | 3 | — | — | — | 0 | 0 | 0 | 0 | 0 | 0 | S̲ | S | 0 | 0 | 0 | 0 | 0 | 0 | 0 | S |
| Tumour cross sectional profile | 4 | — | — | — | — | S̲ | S̲ | S̲ | S̲ | S | 0 | 0 | 0 | 0 | S̲ | S | S̲ | 0 | 0 | S̲ |
| Height of tumour above skin surface | 5 | — | — | — | — | — | S̲ | S̲ | S̲ | S | 0 | 0 | 0 | 0 | S̲ | S | S̲ | 0 | 0 | S |
| Maximum tumour diameter(<10mm or ⩾10mm) | 6 | — | — | — | — | — | — | S̲ | S̲ | S̲ | 0 | 0 | 0 | 0 | 0 | 0 | S | 0 | 0 | 0 |
| Tumour ulceration | 7 | — | — | — | — | — | — | — | S̲ | S̲ | 0 | 0 | 0 | S | S | 0 | S̲ | 0 | S | S̲ |
| Maximum tumour thickness (Breslow) | 8 | — | — | — | — | — | — | — | — | S̲ | 0 | 0 | 0 | 0 | S̲ | S | S̲ | S | S | S |
| Level of invasion (Clark) | 9 | — | — | — | — | — | — | — | — | — | 0 | 0 | 0 | 0 | S | 0 | S̲ | 0 | S̲ | 0 |
| Actinic collagenous degeneration | 10 | — | — | — | — | — | — | — | — | — | — | 0 | S | 0 | 0 | 0 | S | 0 | 0 | 0 |
| Predominant tumour cell type | 11 | — | — | — | — | — | — | — | — | — | — | — | 0 | S | 0 | S | 0 | 0 | 0 | 0 |
| Tumour cell heterogeneity | 12 | — | — | — | — | — | — | — | — | — | — | — | — | 0 | 0 | 0 | 0 | 0 | 0 | 0 |
| Tumour cell nucleoli | 13 | — | — | — | — | — | — | — | — | — | — | — | — | — | S | S | 0 | 0 | 0 | 0 |
| Tumour giant cells | 14 | — | — | — | — | — | — | — | — | — | — | — | — | — | — | S̲ | S̲ | 0 | 0 | 0 |
| Tumour cell pleomorphism | 15 | — | — | — | — | — | — | — | — | — | — | — | — | — | — | — | S | 0 | 0 | 0 |
| Tumour cell mitotic activity | 16 | — | — | — | — | — | — | — | — | — | — | — | — | — | — | — | — | S | S | S |
| Host reaction (cell) strength | 17 | — | — | — | — | — | — | — | — | — | — | — | — | — | — | — | — | — | 0 | 0 |
| Vascular invasion | 18 | — | — | — | — | — | — | — | — | — | — | — | — | — | — | — | — | — | — | 0 |
| Tumour type (Clark) | 19 | — | — | — | — | — | — | — | — | — | — | — | — | — | — | — | — | — | — | — |

**Figure 2** Summary of the clinicopathological correlations found. If the association between a pair of variables was not statistically significant at $P=0.05$ the corresponding entry in the table is 0. If significant at $P<0.05$ the entry is S, and if significant at $P<0.0001$ it is S̲.

"level of invasion", with invasion present in 10% of level III tumours, 28% of level IV, and 67% of level V. The obvious trend being for the incidence of vascular invasion to vary directly with the level of microinvasion. Tumour ulceration showed a highly significant association with seven other parameters including cross-sectional profile, maximum tumour thickness, level of microinvasion, and tumour cell mitotic activity.

*Survival following treatment*

Figure 3 provides actuarial survival rates for the overall group of 150 patients (114 females and 36 males).

Of the 19 potential predictor variables listed in Table III ten were found to be significantly associated with survival following primary operation. Six parameters were associated with survival to ⩾5 years; namely, cross-sectional profile, height of tumour above skin surface, tumour ulceration, sex of patient, maximum tumour thickness, and level of microinvasion (Table IV).

The overall actuarial cumulative proportion of patients surviving to seven years was 71% (78% female and 37% male). The corresponding 5-year rates were 77%, 83%, and 49%, and the corresponding three year rates were 81%, 86%, and 63%. The median survival of the 36 males was 4.8 years. A significant difference was demonstrated between the males and females in cumulative survival to 7 and to 5 years ($P<0.001$), the difference in survival to three years failed to reach the 95% level of confidence.

Figure 4 shows the arcsine transformation of the overall survival data. Two linear regression lines can be drawn to fit the data, one (AB) relating to years 1–3.5 from the primary operation, its

**Table IV** Significant associations between survival and 19 potential clinical and histological predictor variables.

| *Parameter compared* | $\chi^2$ | *df* | *P* |
|---|---|---|---|
| Sex of patient | 12.9400 | 1 | <0.001 |
| Age at first diagnosis | | | |
| (< or ⩾50 years) | 5.1056 | 1 | <0.05 |
| Tumour cross-sectional profile | 9.5598 | 2 | <0.01 |
| Height above skin surface | 8.5686 | 3 | <0.05 |
| Tumour ulceration | 8.1060 | 1 | <0.01 |
| Maximum tumour thickness | 15.6028 | 3 | ~0.001 |
| Level of invasion | 17.4597 | 3 | <0.001 |
| Tumour cell mitotic activity | 13.9636 | 6 | <0.05 |
| Vascular invasion by tumour | 8.0903 | 3 | <0.05 |
| Tumour type | 14.2604 | 3 | <0.05 |

Yates' correction applied where indicated.

**Figure 3** Actuarially calculated survival of: (a) all patients (150); (b) females (114); and (c) males (36). The bar lines indicate ±s.e. The broken line continuation of the curve indicates the number of patients surviving.

steepness of slope reflects the earlier deaths of males. The other line (CD) relating to years 3.5–10 is less steep and represents mainly the survival of females. The estimated hazard functions relative to the three survival functions (overall; male; and female) are depicted in Figure 5 which shows maxima during the second year after operation. A second peak occurs in male patients around the fifth year and in females about the seventh year after primary tumour directed surgery. A third hazard peak for female patients occurs between years 13 and 14.

### *Regression analysis and prediction of survival*

Although logistic regression equations based upon Cox's model were calculated for clinically disease free survival for 3, 5, and 7 years following the primary operation, the regression equation for 4-year survival calculated by the MLR technique was selected as showing the best "goodness of fit" and having the greatest predictive value relative to population tested. Data relative to goodness of fit of this regression equation and the variables selected for inclusion are presented in Table V. Insufficient cases were avilable in the study to allow a logistic regression equation of acceptable goodness of fit to be produced with survival to 7 years as the dependent variable.

Figure 6 presents the percentage of correct classifications as a function of various cut-off points of the computed probabilities of survival relative to the BMD-PLR MLR program with 5 year survival as the dependent variable. Thus the level of probability that an estimated prediction of survival is correct for a given cut-off point can be assessed.

**Table V** Predictor variables selected for the 5-year survival model by the BMD-PLR MLR stepwise logistic regression program (Engelman, 1979).

| *Regression goodness of fit* | *Variables selected for regression* |
|---|---|
| $\chi^2=46.003$ | Microinvasion level (Clark) |
| df=75 | Patient's sex |
| $P=0.997$ | Tumour type (Clark) |
| | Actinic degeneration |
| | Tumour giant cells |
| | Tumour cell pleomorphism |

### Discussion

The crude female:male sex ratio of melanoma patients in the present study was 3.2:1 which, when corrected to the European standard population (Waterhouse *et al.*, 1976) became 2.9:1. This predominance of females as compared with males is common in the majority of melanoma series reported in the British Isles and other European countries but much less common in those reported from centres in the United States. It has long been recognised that this female predominance is due to a higher incidence of MM of the female lower limb and in particular that part of the limb below the knee (Knutson *et al.*, 1971). The high incidence of MM at this site has been attributed by many authors to exposure to ultraviolet irradiation (Lee, 1972; Magnus, 1981), although this view has recently been challenged by Lee & Storer (1980) who

**Figure 4** Weighted arcsine transformation of survival proportion for the whole group of patients studied fitted with linear regression lines by the method of least squares.

**Figure 5** Estimated hazard functions relative to the survival functions illustrated (*Figure 3*) for 150 patients (114 females and 36 males).

**Figure 6** Percentage of correct classifications at various cutpoints on the computed probabilities (BMD-PLR MLR model for 5-year survival).

in an analysis of data published by the WHO found the incidence and mortality of MM in women of reproductive and menopausal age in England and Wales to be higher than the corresponding rates for men. According to these writers the F:M ratio reaches a peak of about 3:1 between the ages of 30–40 years. They suggest that the low rates for environmental tumours enables a hormone dependent variable to reveal itself in the British population.

A significant association was observed between age at first diagnosis and tumour type ($P<0.05$). The findings for LMM being in accord with those of Larsen & Grude (1978), Clark *et al.*, (1975), and McGovern (1970). With SSM the age at diagnosis was roughly equally divided above and below age 50 years, a finding in accord with that of Larsen & Grude (1978). Clark *et al.* (1975) record SSM to have a peak incidence in the fifth decade but with common occurrence in the third, fourth, sixth, and seventh decades. The age at diagnosis for NMM was similar to that recorded by Larsen & Grude (1978) but differed from that of McGovern (1970) in that he found the greatest number of NMM to occur in patients aged <50 years.

The regional distribution of tumour types (Table II) shows findings similar to those of McGovern (1970), Smith (1976) and Larsen & Grude (1978) in that LMM occurred most frequently on the skin of the head and neck. The distribution of SSM is similar to that recorded by Smith (1976) with the greatest incidence occurring on the lower extremity but differs from that of Clark *et al.* (1975) who found the back to be the commonest site for SSM and 31% of all SSM to occur at this site. The regional distribution of NMM is in accord with the findings of Smith (1976) and McGovern (1970) who also found NMM to occur most frequently on the lower extremities. The findings in the present study support the statement of Clark *et al.* (1975) that SSM is the dominant form of the disease in Caucasians.

An essential prelimary before any statistical examination is carried out is to examine the basic data for quality and to determine any significant associations between pairs of potential predictor variables. If some of the variables are significantly correlated, then any one of the correlated variables is likely to be as good a predictor as all of them. If other studies show that a given predictor variable has prognostic value, then it should be retained (Lee, 1980). For this reason the 19 potential predictor variables were examined by contingency table analysis for significant associations. Sixty-six such associations were found ($P \leqslant 0.05$) and of these associations 28 were found to be highly significant ($P \leqslant 0.0001$). The importance of the latter will be found in explaining certain apparent anomalies in the variables selected in the present study for the 5-year survival model and those selected by other reported studies using multivariate analysis. The results of contingency table analysis for significant associations between the 19 predictor variables selected for regression analysis are presented in Figure 2.

Multivariate analysis of the 19 predictor variables using the BMD-PLR MLR computer program, with 5-year survival as the dependent variable, are presented in Table V. The variables selected as dominant in this analysis (microinvasion level (Clark) and patients' sex) differ from those selected in other studies using a similar Cox model or a variant where tumour maximum thickness is shown to be the dominant prognostic variable (e.g. Balch *et al.*, 1979). A possible reason for this apparent anomaly may lie in the fact that contingency table analysis shows these two variables to have a highly significant association ($P \leqslant 0.0001$) and Lee's observation regarding selection of a variable from a group showing a high degree of correlation, may provide a partial explanation for this finding. Day *et al.*, (1982) have also commented on apparent anomalies in the variables selected for the prognostic regression equation and have stressed the importance of examining alternative Cox models. They observe that other combinations of variables may predict outcome as well or better than the primary combination, especially if some predictor variables are highly correlated. They comment that this alternative model phenomenon may explain the apparent disparate results when the Cox model is used to determine prognostic factors for apparently identical groups of patients. Day and his co-workers rightly stress that emphasis has thus shifted from finding the "best" group of variables to finding the combination of variables with the highest concordance. Kopf *et al.* (1981) observe that a knowledge of seemingly disparate findings and the reasons for them may reveal important variations in the biological behaviour of malignant melanoma in widely separate regions of the world. It would also appear possible that the emergence of "sex" as a dominant variable in this study is related to the fact that 81% of the males showed a maximum tumour thickness of 1.5 mm as opposed to 53% of the female.

With regard to the present study it must be emphasized that all patients received surgical treatment according to a standardized protocol, with local wide excision as the treatment of choice unless contraindicated by anatomical consideration. Furthermore elective lymphadenectomy was carried out on only 2% of the patients. Should the treatment of patients vary from the present protocol then "treatment" should be added to the predictor variables analysed and further analyses made.

Finally comparison of the results of this study with those using multivariate analysis of data from other centres would suggest the following: (i) a prognostic regression equation derived from regression analysis at one centre should not be applied for prognostic purposes at a second centre until it has been shown to give an acceptably high proportion of correct predictions at that centre; (ii) if the regression analysis used is based upon the Cox model then investigation of alternative Cox models should be made to select the prognostic equation giving the highest degree of concordance; and (iii) the regression equation used at a given centre for predictive purposes should be periodically checked to ensure that the acceptable proportion of correct predictions is maintained and has not varied as a result of increasing the test population or variations in its composition.

### Addendum

The authors realise that they have not applied the 1972 regression model suggested by Cox [Cox, D.R. (1972) Regression models and life-tables. *J. Royal Stat. Soc. Br.*, **34,** 187]. and that the analyses described in this article do not include a time dependent factor as suggested by Cox. The BMDP package written by the Health Sciences Computing Facility of the University of California was not, however, published until 1979 and incorporates, for example, the 1975 Jenwich & Moore algorithm for estimating coefficients that maximise the likelihood function discussed in Cox's 1972 paper. The PLR program was used because it was available and well proven on the University CYBER installation and because it produced a model closely fitting the 90 cases available for the 5-year survival analysis and the other analyses that were undertaken. Current research is aimed at a comparison of the 2 Cox regressions.

Thanks are due to Prof. R.J. Ord-Smith of the Postgraduate School of Studies in Computing, University of Bradford, for constant help and advice relative to computation. We are grateful to Mr. R. Grimshaw, Chief Medical Laboratory Scientific Officer of the Department of Histopathology, St. Luke's Hospital, Bradford, and to Mr. P. Harrison, Medical Photographer of the same hospital, for expert technical assistance. We gratefully acknowledge the help given by the consultant surgeons, particularly Mr. T.L. Barclay and Mr. D.J. Crockett, who referred material for examination and allowed access to their clinical notes.

### References

BALCH, C.M., SOONG, S-J., MURAD, T.M., INGALLS, A.L. & MADDOX, W.A. (1979). A multifactorial analysis of melanoma. II. Prognostic factors in patients with stage I (localized) melanoma. *Surgery*, **86**, 343.

BEARDMORE, G.L., QUINN, R.L. & LITTLE, J.H. (1970). Malignant melanoma in Queensland: Pathology of 105 fatal cutaneous melanomas. *Pathology*, **2**, 277.

BRESLOW, A. (1970). Thickness, cross-sectional areas and depth of invasion in the prognosis of cutaneous melanoma. *Ann. Surg.*, **172**, 902.

CLARK, W.H. Jr., AINSWORTH, A.M., BERNADINO, E.A., & 3 others. (1975). The developmental biology of primary human malignant melanomas. *Semin. Oncol.*, **2**, 83.

CLARK, W.H. Jr., FROM, L., BERNADINO, E.A. & MIHM, M.C. (1969). The histogenesis and biologic behavior of primary human malignant melanomas of the skin. *Cancer Res.*, **29**, 705.

COCHRAN, A.J. (1968). Method of assessing prognosis in patients with malignant melanoma. *Lancet*, **ii**, 1062.

COX, D.R. (1970). *Analysis of Binary Data*. London: Chapman & Hall.

CUTLER, S.J. & EDERER, F. (1958). Maximum utilization of the life table method in analysing survival. *J. Chron. Dis.*, **8**, 699.

DAY, C.L. Jr., LEW, R.A., MIHM, M.C. Jr., & 19 others. (1982). A multivariate analysis of prognostic factors for melanoma patients with lesions $\geq$3.65 mm in thickness. *Ann. Surg.*, **195**, 44.

ENGELMAN, L. (1979). BMDP—PLR stepwise logistic regression. In *Biomedical Computer Programs, P series*. (Eds. Dixon & Brown). Los Angeles: University of California Press.

HARDMEIER, Th., NUSSBAUMER, U. & KOTNIK, G. (1968). Zur Prognostischen bedeutung histologischer kriterien beim malignen melanom. *Virchows Arch. Pathol. Anat.* (Abt. A) **345**, 23.

KNUTSON, C.O., HORI, J.M. & SPRATT, J.S. (1971). Melanoma. *Current Problems Surg.* **12**, 1.

KOPF, A.W., RIGEL, D., BART, R.S. & 7 others. (1981). Factors related to thickness of melanoma. Multifactorial analysis of variables correlated with thickness of superficial spreading malignant melanoma in man. *J. Dermatol. Surg. Oncol.*, **7**, 645.

LARSEN, T.E. & GRUDE, T.H. (1978). A retrospective histological study of 669 cases of primary cutaneous malignant melanoma in clinical stage I: Part 1. *Acta Pathol. Microbiol. Scand.*, (Sect. A) **86**, 437.

LEE, E.T. (1980). *Statistical Methods for Survival Data Analysis*. Belmont, California: Lifetime Learning Publications. pp. 299, 425.

LEE, J.A.H. (1972). Sunlight and the etiology of malignant melanoma. In *Melanoma and Skin Cancer. Proceedings of the International Cancer Conference, Sydney 1972*. Sydney: V.C.N. Blight, Government Printer.

LEE, J.A. & STORER, B.E. (1980). Excess of malignant melanomas in women in the British Isles. *Lancet*, **ii**, 1337.

MAGNUS, K. (1981). Habits of sun exposure and risk of malignant melanoma: An analysis of incidence rates in Norway 1955–1977 by cohort, sex, age, and primary tumor site. *Cancer*, **48**, 2329.

McGOVERN, V.J. (1970). The classification of melanoma and its relationship with prognosis. *Pathology*, **2**, 85.

McGOVERN, V.J., MIHM, M.C. Jr., BAILLY, C. & 9 others. (1973). The classification of malignant melanoma and its histologic reporting. *Cancer*, **32**, 1446.

POLK, J.C., Jr. & LINN, B.S., (1971). Selective regional lymphadenectomy for melanoma: A mathematical aid to clinical judgement. *Ann. Surg.*, **174**, 402.

SMITH, J.L. Jr. (1976). Histopathology and biologic behavior of malignant melanoma. In: *Neoplasms of the Skin and Malignant Melanoma*. (Eds. Freitag & Culhane). Chicago: Year Book Medical Publishers. p. 293.

WATERHOUSE, J., MUIR, C., CORREA, P. & POWELL, J., Eds. (1976). *Cancer Incidence in Five Continents, Vol. III*. Lyon: IARC Publications.